# The Role of ERBB Signaling Pathway-Related Genes in Kidney Renal Clear Cell Carcinoma and Establishing a Prognostic Risk Assessment Model for Patients

**DOI:** 10.3389/fgene.2022.862210

**Published:** 2022-07-12

**Authors:** Zicheng Wang, Jiayi Li, Peizhi Zhang, Leizuo Zhao, Bingyin Huang, Yingkun Xu, Guangzhen Wu, Qinghua Xia

**Affiliations:** ^1^ Department of Urology, Shandong Provincial Hospital Affiliated to Shandong First Medical University, Jinan, China; ^2^ Medical Science and Technology Innovation Center, Shandong First Medical University and Shandong Academy of Medical Sciences, Jinan, China; ^3^ School of Business, Hanyang University, Seoul, South Korea; ^4^ Department of Urology, Shandong Provincial Hospital, Cheeloo College of Medicine, Shandong University, Jinan, China; ^5^ Department of Urology, Dongying People’s Hospital, Dongying, China; ^6^ Department of Pathology, The First People’s Hospital of Zhoukou, Zhoukou, China; ^7^ Department of Endocrine and Breast Surgery, The First Affiliated Hospital of Chongqing Medical University, Chongqing, China; ^8^ Department of Urology, The First Affiliated Hospital of Dalian Medical University, Dalian, China

**Keywords:** TCGA, KIRC, ERBB signaling pathway, pan-cancer, GDSC

## Abstract

**Objective:** We aimed to investigate the potential role of ERBB signaling pathway–related genes in kidney renal clear cell carcinoma (KIRC) and establish a new predictive risk model using various bioinformatics methods.

**Methods:** We downloaded the KIRC dataset and clinicopathological information from The Cancer Genome Atlas database. Univariate Cox analysis was used to identify essential genes significantly associated with KIRC progression. Next, we used the STRING website to construct a protein–protein interaction network of ERBB signaling pathway–related molecules. We then used the least the absolute shrinkage and selection operator (LASSO) regression analysis to build a predictive risk model for KIRC patients. Next, we used multiple bioinformatics methods to analyze the copy number variation, single-nucleotide variation, and overall survival of these risk model genes in pan-cancer. At last, we used the Genomics of Drug Sensitivity in Cancer to investigate the correlation between the mRNA expression of genes associated with this risk model gene and drug sensitivity.

**Results:** Through the LASSO regression analysis, we constructed a novel KIRC prognosis–related risk model using 12 genes: SHC1, GAB1, SOS2, SRC, AKT3, EREG, EIF4EBP1, ERBB3, MAPK3, transforming growth factor-alpha, CDKN1A, and PIK3CD. Based on this risk model, the overall survival rate of KIRC patients in the low-risk group was significantly higher than that in the high-risk group (*p* = 1.221 × 10^−15^). Furthermore, this risk model was associated with cancer metastasis, tumor size, node, stage, grade, sex, and fustat in KIRC patients. The receiver operating characteristic curve results showed that the model had better prediction accuracy. Multivariate Cox regression analysis showed that the model’s risk score was an independent risk factor for KIRC. The Human Protein Atlas database was used to validate the protein expression of risk model–associated molecules in tumors and adjacent normal tissues. The validation results were consistent with our previous findings.

**Conclusions:** We successfully established a prognostic-related risk model for KIRC, which will provide clinicians with a helpful reference for future disease diagnosis and treatment.

## Introduction

Renal cell carcinoma (RCC) is the most common urinary system tumor, and its incidence rate is increasing annually (Qi et al., 2021). RCC is the most common primary renal malignancy, accounting for 90–95% of all renal cancer cases (Xu et al., 2020b). Although the detailed mechanism and etiology of RCC have yet to be fully elucidated, its incidence rate may be related to smoking, hypercholesterolemia, occupational contact carcinogens, and genetic factors (Che et al., 2021). The main treatment methods for RCC include surgery, chemotherapy, and immunotherapy. However, approximately one-third of RCC patients still have distant metastasis (Gupta et al., 2008). Metastatic RCC exhibits obvious drug resistance to immunotherapy and radiotherapy due to the high dynamics, adaptability, and heterogeneity of the tumor microenvironment (Lai et al., 2021). Therefore, identifying new treatment options for RCC is highly necessary. There are multiple subtypes of RCC, and approximately 70% of patients are diagnosed with clear cell renal cell carcinoma (ccRCC), also known as kidney renal clear cell carcinoma (KIRC) (Jonasch et al., 2021). With the application of targeted drugs for KIRC, an increasing number of patients with advanced RCC have achieved better therapeutic effects. However, these patients still have several issues with the treatment progress, such as drug resistance (Makhov et al., 2018). This is because the occurrence of tumors is a complex process that is not caused by the activation of a single proto-oncogene or the imbalance of tumor suppressor genes. This may be caused by the activation or imbalance of multiple biological pathways (Porporato et al., 2018). Therefore, this principle is used to research the role of the entire pathway in KIRC, understand the pathogenesis of KIRC, and explore new treatments.

ERBB tyrosine kinase family members share some common gene changes in cancer. Through gene changes, abnormally activated tyrosine kinases can promote tumor occurrence, growth, and development. More importantly, abnormal signals of ERBB family members play an essential role in tumorigenesis and evasion of antitumor immunity in many tumors (Kumagai et al., 2021). Evidence shows that the immune response is critical in KIRC (Xu et al., 2019). Therefore, we hypothesized that the ERBB signaling pathway plays a vital role in the occurrence and development of KIRC. The type I subclass of the receptor tyrosine kinase family consists of ERBB or epidermal growth factor receptors (EGFRs), including ERBB1/HER1, ERBB2/HER2, ERBB3/HER3, and ERBB4/HER4 (Hynes, 2007). ERBB receptors are activated *via* homodimerization or heterodimerization. The ERBB family is unique among various receptor tyrosine kinases; ERBB3 has impaired kinase activity, whereas ERBB2 has no direct ligand. Therefore, heterodimerization is an important mechanism that allows all ERBB receptors to be activated by ligand stimulation. The activated ERBB receptor binds to many signaling molecules and activates related signaling pathways (Yarden and Pines, 2012). In cancer, abnormal activation of EGFR and HER2 can be induced by gene amplification, point mutation, deletion, and autocrine ligand–receptor stimulation (Sharma and Settleman, 2009). These gene mutations abnormally activate EGFR/ERBB1 and ERBB signals and are independent of ligand–receptor stimulation, resulting in the occurrence and development of tumors. Owing to its limited kinase activity, the carcinogenic function of ERBB3 is largely mediated by its overexpression and interaction with EGFR/ERBB1 or ERBB2 (Jaiswal et al., 2013). The role of ERBB4 in tumor development is inconsistent because its proto-oncogene and tumor suppressor gene subtypes have different activities. EGFR/ERBB1 is mainly associated with lung adenocarcinoma and squamous cell carcinoma development. ERBB2 is abnormally activated in extensive changes, especially in breast cancer, glioblastoma, and non-small cell lung cancer (Bargmann et al., 1986). ERBB3 is closely associated with ovarian, gastrointestinal, and bladder tumors (Jaiswal et al., 2013). ERBB4 regulates the occurrence and development of lung cancer and metastatic melanoma (Roskoski, 2014). According to research on the relationship between ERBB and its signaling pathway in tumors, it is expected to become a target for cancer treatment.

The genes associated with the ERBB pathway in KIRC were investigated in the current study, and most showed significant expression differences. By conducting least the absolute shrinkage and selection operator (LASSO) regression analysis, we found that most genes in the ERBB pathway play a crucial role in KIRC. At the same time, 12 ERBB pathway–related genes were constructed into a KIRC prognostic risk model. Better prediction accuracy of the model is shown by the receiver operating characteristic (ROC) curve. In addition, increasing evidence shows that the occurrence and development of tumors are strongly correlated with immune infiltration. We explored the relationship between related genes and immune infiltration. Our results provide a new approach to clinical diagnosis and treatment for KIRC patients.

## Materials and Methods

### Data Acquisition

In November 2021, KIRC mRNA expression data and clinical datasets were obtained from The Cancer Genome Atlas (TCGA) database (https://portal.gdc.cancer.gov/). The KIRC dataset in TCGA database includes 72 normal samples and 539 KIRC samples. The ERBB pathway was found in the gene set enrichment analysis (GSEA) database (https://www.gsea-msigdb.org/gsea/index.jsp), and the genes in this pathway were evaluated (Mootha et al., 2003; Subramanian et al., 2005). The path was named KEGG_ERBB_SIGNALING_PATHWAY, and the systematic path was named M12775. In addition, to verify whether the risk model can be applied to other databases, we obtained RNAseq data and corresponding clinical information of 136 RCCs from the ICGC database (https://dcc.icgc.org/releases/current/Projects). We used multivariate Cox regression analysis to construct a predictive model, log-rank was used to test the KM survival analysis to compare the survival differences between the aforementioned two or more groups, and timeROC analysis was performed to judge the accuracy of the prediction model.

### Data Processing and Analysis

The Perl language was used to organize and transform the data, combined with a powerful manipulation software, namely, the R software, for statistical analysis and graphing. Heatmaps were drawn by manipulating the “pheatmap” package and performing statistical analysis by running the “limma” package. In addition, we performed LASSO regression curve analysis for ERBB signaling pathway–related genes in KIRC using the “glmnet” and “survival” packages. Afterward, the Kaplan–Meier survival “survival” package was used to draw survival curves, and the “ROC” package was used to draw ROC curves. At last, we performed univariate and multivariate Cox analyses based on this risk model.

### GEPIA Website

The GEPIA website integrates TCGA cancer big data and GTEx normal tissue big data using bioinformatics technology to solve significant problems in cancer biology, revealing cancer subtypes, driver genes, alleles, and differentially expressed or carcinogenic factors to dig deeper into novel cancer targets and markers (http://gepia2.cancer-pku.cn/#index) (Tang et al., 2017). We utilized the GEPIA database to investigate the overall survival of ERBB pathway–related genes in various tumors.

### Gene Set Cancer Analysis Website

The Gene Set Cancer Analysis website integrates cancer genome data from TCGA for 33 cancer types, drug response data from the Genomics of Drug Sensitivity in Cancer (GDSC) and the Cancer Therapeutics Response Portal, and normal tissue data from GTEx for the dynamic and visual analysis of cancer genomes (http://bioinfo.life.hust.edu.cn/web/GSCALite/) (Liu et al., 2018). In addition, gene set analysis can be performed using the unified data analysis pipeline of the database. We used this database to study gene mutation levels, methylation levels, and immune cell infiltration of ERBB signaling pathway–related risk model genes in various tumors. Furthermore, the database also analyzed the relationship between the risk model genes related to the ERBB signaling and tumor pathways.

### ImmuCellAI Website

ImmuCellAI, a network platform for the comprehensive analysis of immune cell abundance, estimates the infiltration abundance of 24 immune cells based on gene expression datasets, including RNAseq and microarray data (http://bioinfo.life.hust.edu.cn/ImmuCellAI/). At the same time, ImmuCellAI can predict the response of patients to immune checkpoint inhibitor therapy. The 24 immune cells of ImmuCellAI consist of 18 T-cell subtypes and six other immune cells: B cells, natural killer cells, monocytes, macrophages, neutrophils, and dendritic cells (Miao et al., 2020). Based on the established risk model associated with the ERBB signaling pathway, we analyzed the infiltration of 24 types of immune cells in various cancers. We then used the R software to draw the corresponding heatmap for visual analysis. Spearman’s correlation coefficient was used for the statistical analysis.

### TIMER Website

TIMER2.0 (http://timer.cistrome.org/) was used to provide a more accurate level of immune infiltration for the cancer genome map or tumor contour supplied by researchers. At the same time, each module can be used to study the relationship between immune infiltration and clinical characteristics and the relationship with cancer in TCGA cohort. Each module can generate a functional heatmap table (Li et al., 2020). We conducted a more in-depth exploration using the TIMER database to further understand the correlation between ERBB signaling pathway–related risk model genes in KIRC and various immune cell infiltrations. We displayed it in the form of a heatmap using the R software.

### Genomics of Drug Sensitivity in Cancer Database

GDSC contains screening data for 1,000 human cancer cell lines and anticancer drugs. In particular, it includes drug information, omics information of cell lines, and the drug response (IC50) of cancer cell lines. The cell lines in the database generally have typical genetic characteristics and have been widely used for anticancer drug screening. Anticancer drugs include clinically used chemotherapeutic drugs, targeted drugs, and potential cancer treatment drugs (https://www.cancerrxgene.org/) (Rahman et al., 2019). We used this database to explore the sensitivity of ERBB signaling pathway–related risk model genes and various anticancer drugs and plot the corresponding heatmap display.

### The Human Protein Atlas Database

The Human Protein Atlas (HPA) database provides tissue and cellular distribution information for all 24,000 human proteins and is freely available for public inquiries (http://www.proteinatlas.org/) (Thul et al., 2017). The tissue and cellular expression levels of many human proteins can be found in this database. We used this database to explore the protein expression levels of risk model genes associated with the ERBB signaling pathway in normal renal and KIRC tissues.

### Collection of Clinical Tissue Samples

Between January and April 2022, we collected KIRC tumors and adjacent normal tissue samples from six patients undergoing radical nephrectomy at Shandong Provincial Hospital. This study was approved by the ethics committee of Shandong Provincial Hospital. All patients signed an agreement allowing their tissue samples and other clinical information to be available for research purposes.

### Total RNA Extraction and Quantitative Reverse Transcription-Polymerase Chain Reaction Experiments

Total RNA was extracted from tissue samples using the TRIzol reagent (Thermo Fisher Scientific, Waltham, MA, United States) according to the manufacturer’s instructions and then reverse-transcribed into cDNA using the PrimeScript RT reagent (Takara, Japan). At last, qRT-PCR was performed using the SYBR Premix Ex Taq reagent (Takara, Japan).

### Statistical Analyses

In the current study, we compared the differences in the expression of ERBB pathway–related genes in KIRC tumor tissues and adjacent normal tissues *via* one-way ANOVA. The student’s *t*-test was used to estimate the expression differences of ERBB pathway–related genes for different pathological features in the KIRC dataset. The “survminer” package was used to determine the cutoff value of each risk score in the tumor group, and the patients were divided into high- and low-risk groups. The R Studio package was used for statistical analyses. Statistical significance was set to *p* < 0.05.

## Results

### Expression of ERBB Signaling Pathway–Related Genes in KIRC and Univariate Cox Analysis

**FIGURE 1:**
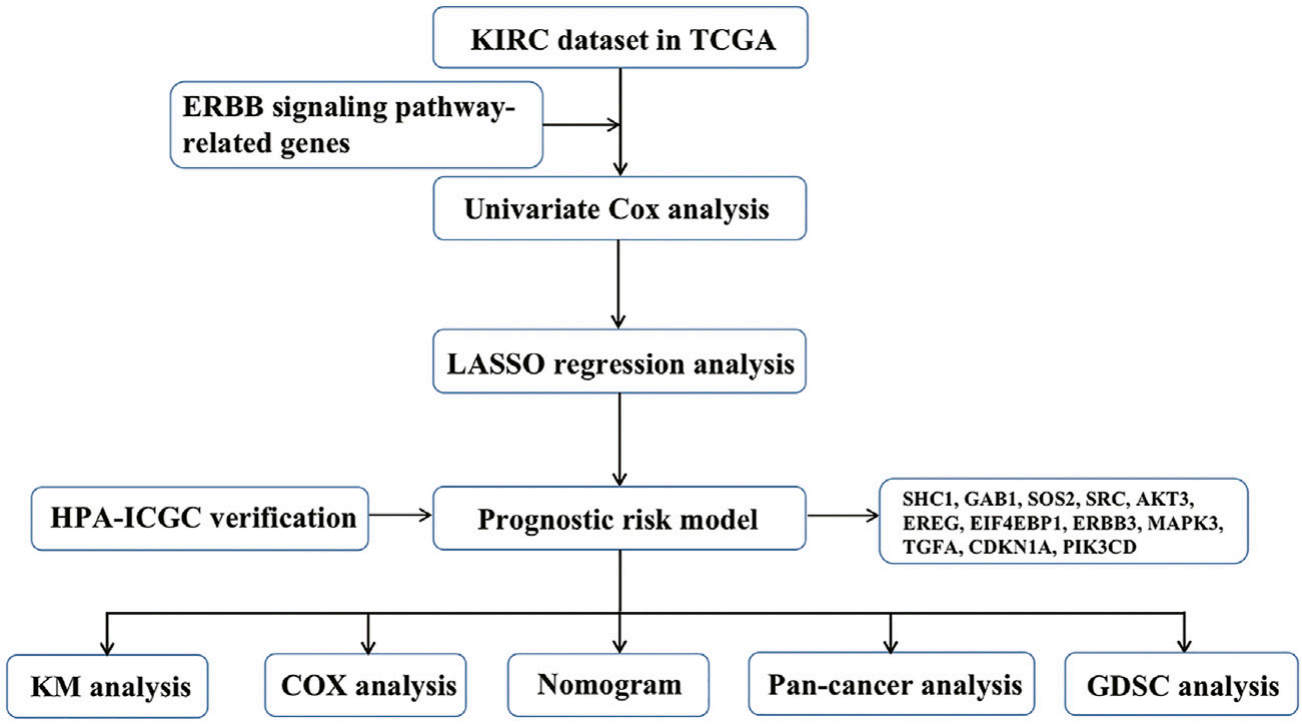
Flow chart of this study.

**FIGURE 2:**
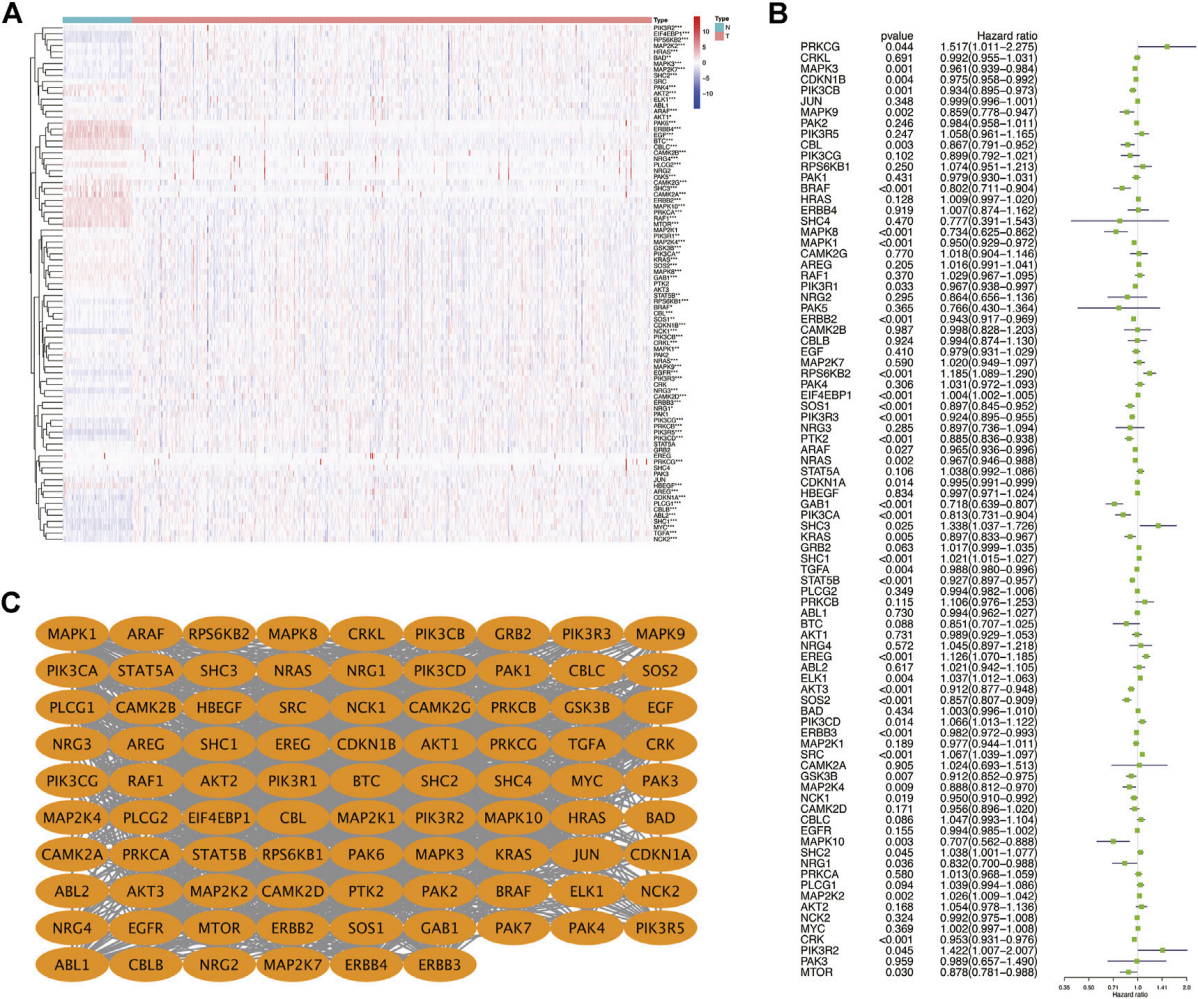
**(A)** Heatmap showing the expression of ERBB signaling pathway-related genes in KIRC. **(B)** Dendrogram showing the results of the univariate Cox analysis of ERBB signaling pathway-related genes in KIRC. **(C)** PPI network showing the interactions and correlations between ERBB pathway molecules. *p < 0:05; **p < 0:01; ***p < 0.001.

First, we drew the corresponding flow chart (Figure 1) to clearly show the research process. Next, we generated heatmaps to study ERBB pathway–associated gene expression in KIRC (Figure 2A). We observed significant differences in most related genes in the ERBB pathway between cancer tissues and paracancerous tissues in the heatmap. Therefore, we can infer that it can change due to changes in the ERBB pathway in the process of tumor occurrence and development. Next, the ERBB pathway–associated genes in KIRC were analyzed *via* univariate Cox regression analysis (Figure 2B). The results showed that the risk ratio of ERBB pathway–related genes had a 95% confidence interval and *p*-value. The results demonstrated that genes encoding mitogen-activated protein kinase 3 (MAPK3), cyclin-dependent kinase inhibitor 1B (CDKN1B), phosphatidylinositol-4,5-bisphosphate 3-kinase catalytic subunit beta (PIK3CB), MAPK9, Cbl proto-oncogene (CBL), B-Raf proto-oncogene (BRAF), MAPK8, MAPK1, phosphoinositide-3-kinase regulatory subunit 1 (PIK3R1), Erb-B2 receptor tyrosine kinase 2 (ERBB2), SOS Ras/Rac guanine nucleotide exchange factor 1 (SOS1), PIK3R3, protein tyrosine kinase 2 (PTK2), A-Raf proto-oncogene (ARAF), NRAS, CDKN1A, and GRB2-associated binding protein 1 (GAB1), PIK3CA, and KRAS, transforming growth factor-alpha (TGFA), signal transducer and activator of transcription 5B (STAT5B), AKT3, SOS2, and ERBB3, glycogen synthase kinase 3 beta (GSK3B), MAP2K4, NCK adaptor protein 1 (NCK1), MAPK10, neuregulin 1 (NRG1), CRK, and mechanistic target of rapamycin kinase were associated with good survival in KIRC patients. To study the interaction between related genes in the ERBB pathway, we used the STRING online database to map the corresponding protein–protein interaction (PPI) network and the Cytoscape tool to identify the PPI network (Figure 2C). The PPI network diagram showed a close interaction between the genes involved in the ERBB pathway.

### Construction of a Novel Prognostic-Related Survival Model in KIRC

**FIGURE 3:**
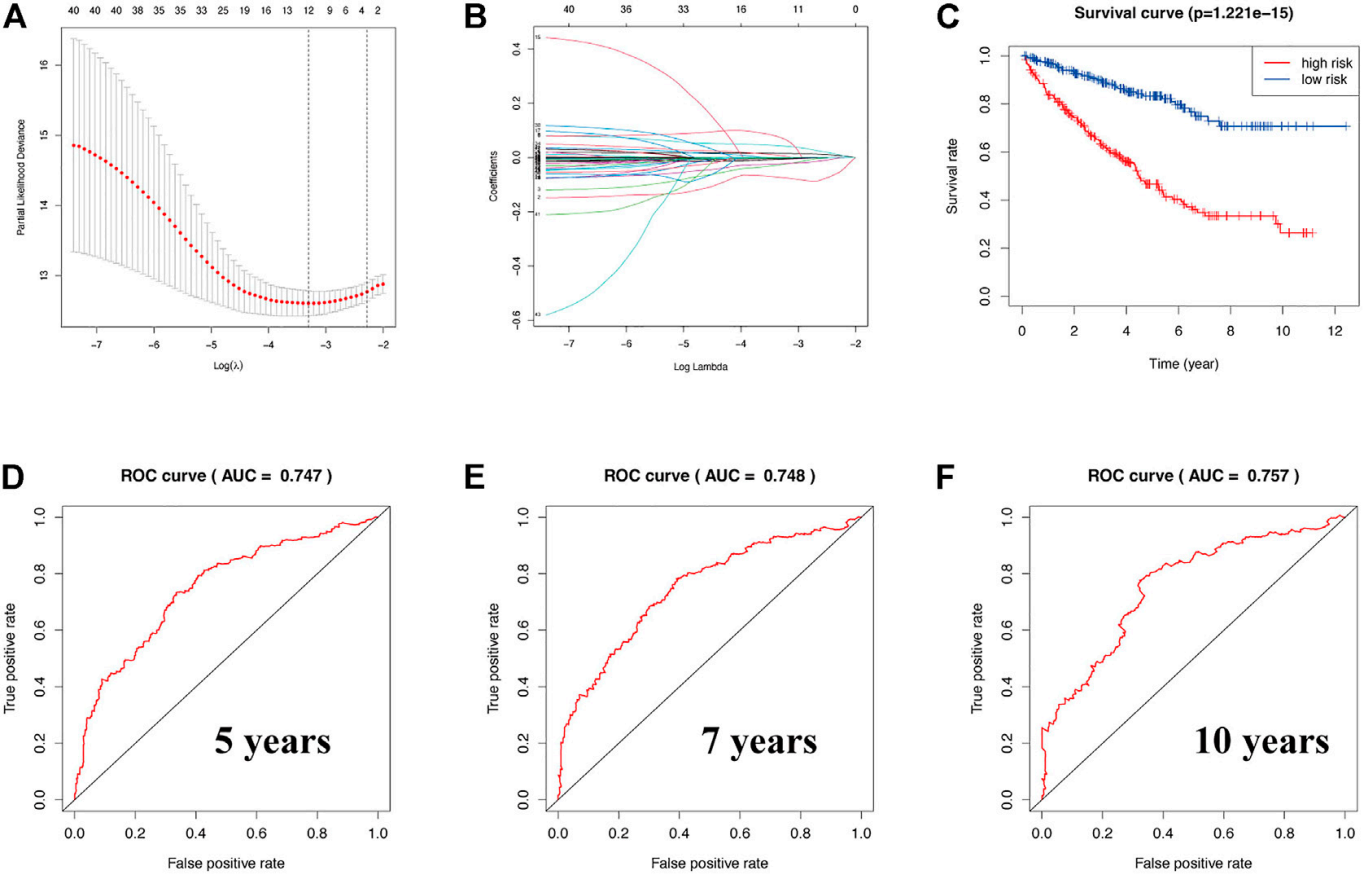
**(A,B)** Construction of a prognostic-related risk model in KIRC using LASSO regression. **(C)** Survival curve based on this model. Red and blue indicate high- and low-risk groups, respectively. **(D–F)** The 5-year AUC was 0.747, the 7-year AUC was 0.748, and the 10-year AUC was 0.757. ROC curve of 5, 7, and 10 years. AUC value greater than 0.7 indicates that the model has more accurate prediction accuracy.

To explore whether a prognostic-related risk model can be constructed in KIRC using ERBB signaling pathway–related genes, we conducted an in-depth exploration using LASSO regression curve analysis (Figures 3A,B). During this process, we created a 12-gene risk model that included 12 molecules: SHC1, GAB1, SOS2, SRC, AKT3, epiregulin (EREG), eukaryotic translation initiation factor 4E binding protein 1 (EIF4EBP1), Erb-B2 receptor tyrosine kinase 3 (ERBB3), MAPK3, TGFA, CDKN1A, and PIK3CD. We then used this prognostic model to classify KIRC patients into two risk groups. From the survival curve, the overall survival rate of KIRC patients in the low-risk group was significantly higher than that in the low-risk group (*p* = 1.221 × 10–15) (Figure 3C). Next, the prognostic prediction performance of this survival model was validated in KIRC patients by analyzing the ROC curves. From the ROC curve analysis, we obtained a 5-year area under the curve (AUC) value of 0.747 (Figure 3D), a 7-year AUC value of 0.748 (Figure 3E), and a 10-year AUC value of 0.757 (Figure 3F), indicating that the model can accurately predict 5-, 7-, and 10-year survival in KIRC patients. The formula for calculating the risk model is as follows:

ERBB Risk Signature = 0.0153348967199206 × SHC1 + 0.0430172526487226 × SRC + 0.0736183869698676 × EREG +0.000614229224522567 × EIF4EBP1 + 0.006822712 × PIK3CD − 0.0733341107404134 × GAB1 − 0.0265211809527625 × SOS2 − 0.0230517422131536 × AKT3 − 0.007240958 × ERBB3 − 0.006311165 × MAPK3 − 0.001331008 × TGFA −0.003804552 × CDKN1A.

### Relationship Between the Risk Model and Clinicopathological Characteristics and Plotting the Corresponding Nomogram in KIRC

**FIGURE 4:**
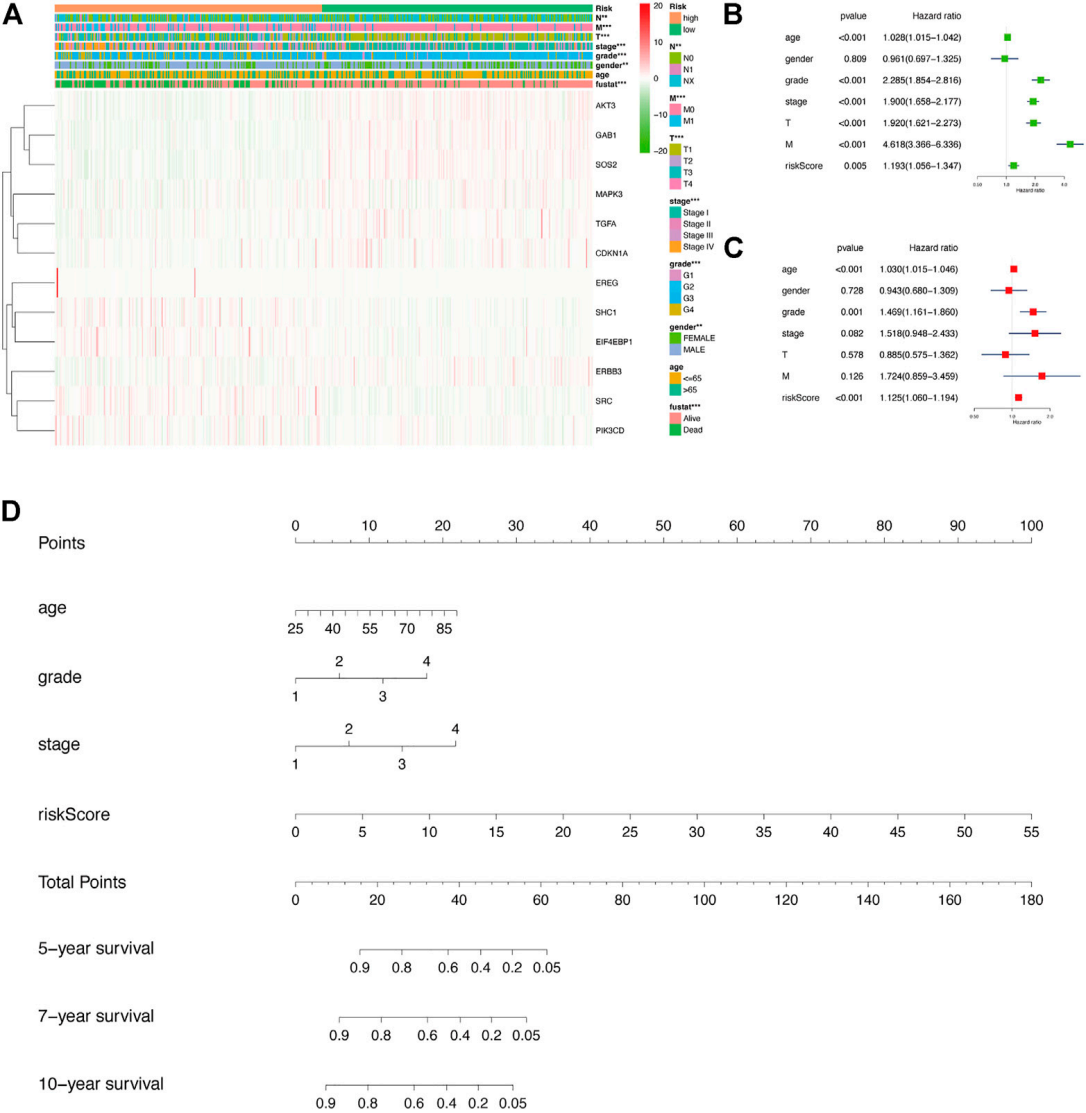
In-depth exploration of the clinical significance of this risk model in KIRC. **(A)** Heatmap showing the relationship between risk scores and clinicopathological features of KIRC. **p < 0.01, ***p < 0.001. **(B,C)** Results of univariate and multivariate Cox regression analyses depicting the correlation among age, sex, grade, stage, tumor size (T), metastasis (M), risk score, and prognosis in KIRC patients. **(D)** The corresponding nomogram based on the risk model, which predicts 5-, 7-, and 10-year overall survival in KIRC patients. Among them, the total points in the sixth row are the sum of scores of each item in the second row to the fifth row.

Next, we generated a heatmap between relevant clinical data and model genes to study the relationship between prognosis risk models and clinicopathological features (Figure 4A). The risk model was associated with cancer metastasis (M), tumor size (T), node (N), stage, grade, sex, and fustat. The patients in the low-risk group typically had a lower histological grade and clinical stage. In addition, we investigated the relationship between KIRC patient prognosis and multiple clinicopathological features *via* a univariate Cox regression analysis (Figure 4B). The corresponding forest plot showed that the overall survival rate of patients was related to the age, grade, stage, T, M, and risk score. Next, using multivariate Cox regression analysis, we found that tumor grade and risk score were independent risk factors associated with overall survival in KIRC patients (Figure 4C). Through two different regression analyses, we observed that the risk score of this model could be used as an excellent prognostic feature for KIRC patients. Afterward, in the nomogram generated based on the risk model, the second to ninth rows represent the age, grade, stage, risk score, total points, and 5-, 7-, and 10-year survival rates of KIRC patients, respectively (Figure 4D).

### Overall Survival and Variation of Model Genes in Pan-Cancer

**FIGURE 5:**
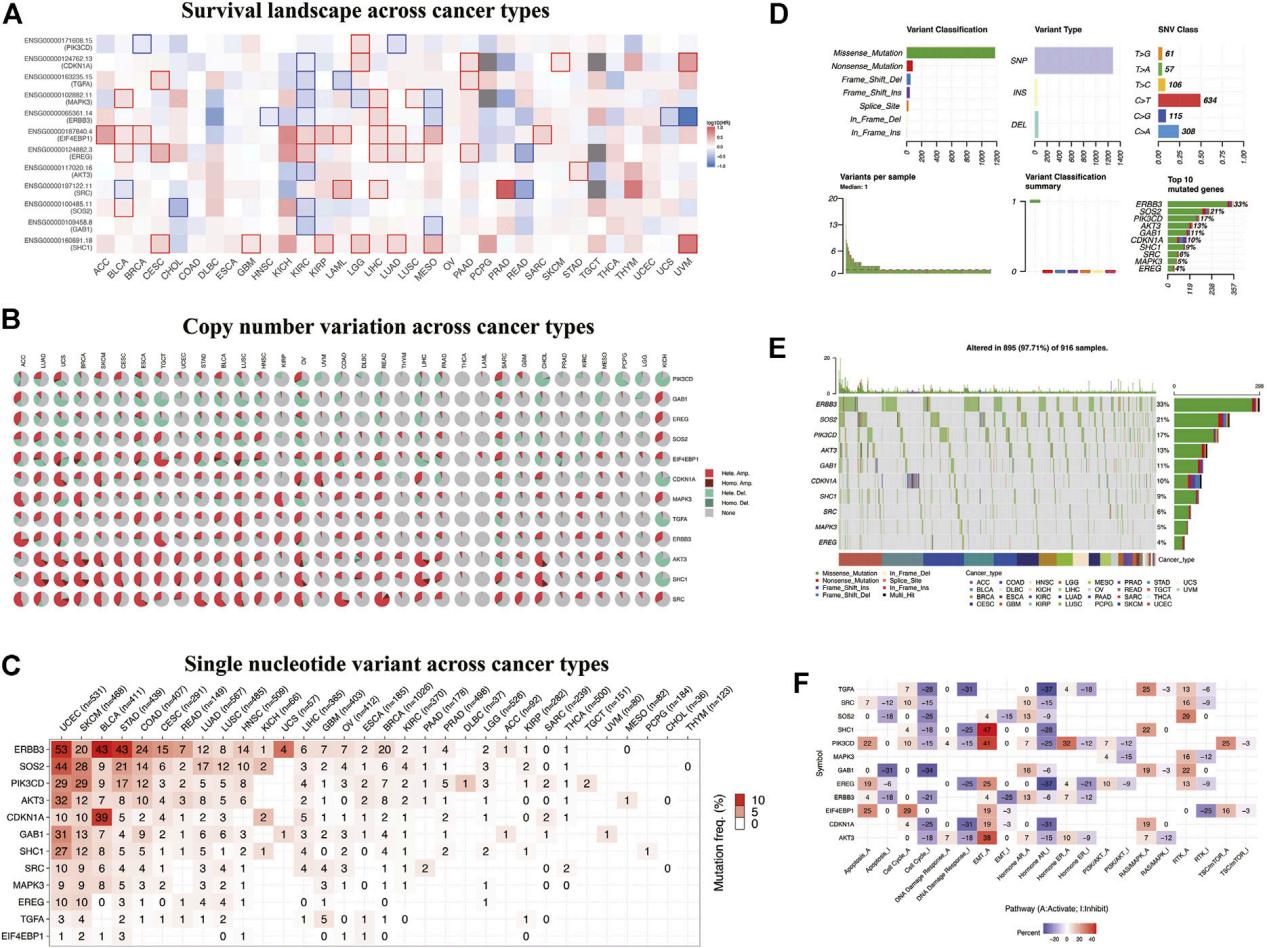
Overall survival and variation analyses of the risk model genes in pan-cancer. **(A)** Heatmap shows the survival landscape across cancer types. The color code bar on the right side shows the corresponding value of log10 (HR). Red and blue represent positive and negative correlations, respectively. **(B)** Heatmap showing copy number variation across cancer types. Light red Hete. Amp. represents heterozygous amplification; light green Hete. Del., represents heterozygous deletion; dark red *Homo*. Amp., represents homozygous amplification; dark green *Homo*. Del., represents homozygous deletion; and gray represents no CNV. **(C–E)** Heatmap showing SNV across cancer types. Color on the square trend from white to red as the frequency of mutation increases. **(F)** Heatmap showing correlations between these risk model genes and biological pathways. Red and blue represent the positive and negative correlations, respectively.

To study the significance of ERBB signaling pathway–related risk genes in pan-cancer, we analyzed the correlation between the overall survival rate of patients with various tumors in the TCGA database and the expression of the risk model genes, followed by the construction of an overall survival heatmap of these genes (Figure 5A). The square with a solid line on the map indicates statistical significance. High expression of CDKN1A, TGFA, MAPK3, ERBB3, AKT3, SOS2, and GAB1 is associated with a better prognosis in KIRC patients. EIF4EBP1 and EREG function as oncogenes in the malignant progression of KIRC. Furthermore, EIF4EBP1 acts as an oncogene in adenoid cystic carcinoma, bladder urothelial carcinoma, breast cancer (BRCA), KIRC, acute myeloid leukemia, liver hepatocellular carcinoma, lung adenocarcinoma, mesothelioma, and sarcoma. Next, we retrieved copy number variants (CNV) and single-nucleotide variants (SNV) data for 32 tumors from TCGA database. We visualized and displayed the variant data in the R software (Figures 5B,C). From Figure 5B, we can see that these 12 risk model genes had higher CNVs in the uterine carcinosarcoma, BRCA, esophageal carcinoma, and ovarian cancer. Figure 5C shows the SNVs of the 12 risk model genes in the different cancer types. The higher the mutation frequency, the darker the red color. ERBB3 had higher SNVs in uterine corpus endometrial carcinoma (UCEC), bladder urothelial carcinoma, and stomach adenocarcinoma, whereas SOS2 had higher SNVs in UCEC and skin cutaneous melanoma. Furthermore, ERBB3, SOS2, PIK3CD, AKT3, and GAB1 had extensive SNVs in the UCEC. ERBB3 had up to 33% of pan-cancer mutations. As a critical gene in the ERBB signaling pathway, we speculated that ERBB3 plays an important role in carcinogenesis (Figures 5D,E). We explored the correlation between these risk model genes and multiple critical biological pathways during carcinogenesis (Figure 5F). We found that SHC1, PIK3CD, and AKT3 could activate epithelial-to-mesenchymal transition (EMT), a vital biological process.

### Immune Infiltration, Methylation, and Drug Sensitivity of Model Genes in Pan-Cancer

**FIGURE 6:**
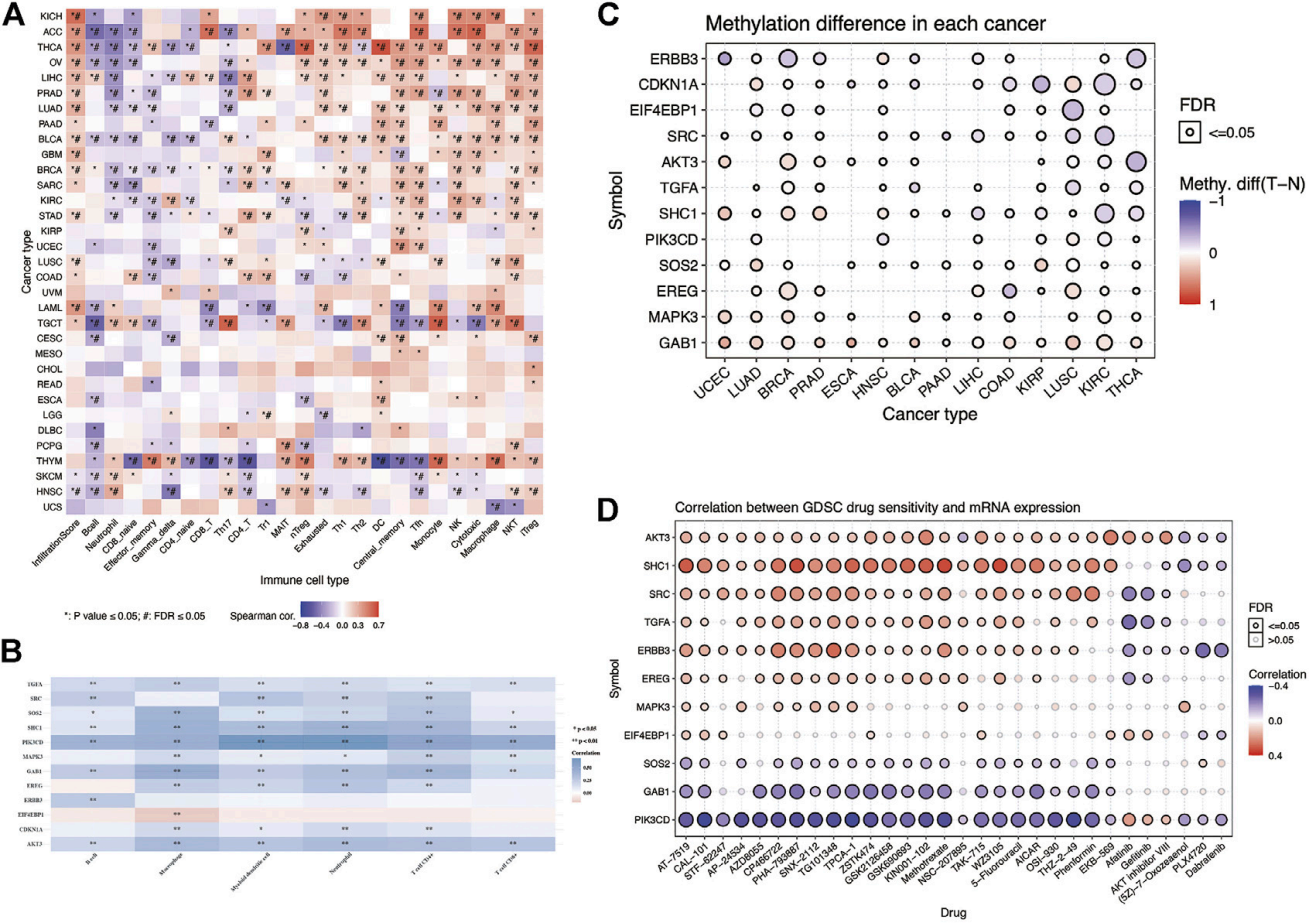
Immune infiltration, methylation, and drug sensitivity analyses of the risk model genes in pan-cancer. **(A)** Heatmap showing the association between the risk model genes and 24 types of immune cell infiltration in pan-cancer. *p-value ≤ 0.05, #FDR ≤0.05. **(B)** Heatmap showing the association between the risk model genes and six major immune cell infiltrations in KIRC. Blue and red represent the positive and negative correlations, respectively. *p < 0.05, **p < 0.01. **(C)** Heatmap showing the methylation differences of the risk model genes in pan-cancer. Red and blue indicate the positive and negative correlations, respectively. **(D)** Heatmap showing the correlation between the GDSC database drug susceptibility and the mRNA expression of the risk model genes. Red and blue indicate the positive and negative correlations, respectively.

ERBB family molecules may play an essential role in evading antitumor immune response (Gainor et al., 2016; Huang and Fu, 2019; Sugiyama et al., 2020). Based on the ImmuCellAI database, we used the R software to visualize the correlation of these risk model genes with 24 immune cell infiltrations in pan-cancer and used Spearman’s correlation coefficient for statistical analysis (Figure 6A). These results indicated that thyroid carcinoma and thymoma induced extensive immune cell infiltration. We observed that these risk model genes were positively correlated with tumor immune cells, such as Tfh, NK, cytotoxic, and NKT, in KIRC. To further explore the correlation between these risk model genes and major immune cell infiltration in KIRC, we analyzed the correlation between 12 risk model genes and six major tumor immune cells using the TIMER database (Figure 6B). We found that TGFA, SHC1, PIK3CD, GAB1, and AKT3 were negatively correlated with six tumor immune cells in KIRC, and EIF4EBP1 was positively correlated with macrophage infiltration. At the same time, we analyzed the methylation differences of these 12 risk model genes between tumor and normal tissues. The results showed significant differences in the methylation of these genes between the normal and tumor tissues (Figure 6C). The larger and darker red the bubbles in the figure, the higher the degree of methylation in the tumor tissue than in the normal tissue. As shown in the figure, the methylation degree of EREG in BRCA is higher than that in the normal tissue, whereas the methylation degree of CDKN1A in KIRC is lower than that in the normal tissue. In particular, to study the link between the mRNA expression of 12 risk model genes and drug sensitivity, we downloaded a variety of drug sensitivity data from the GDSC database, combined the mRNA expression of these 12 risk model genes, and analyzed the 12 risk models. The relationship between model gene expression and drug sensitivity and a corresponding heatmap was drawn using the R software (Figure 6D).

We observed that the mRNA expression of ERBB3 is positively correlated with the ATM kinase inhibitor CP466722. The higher the mRNA expression level of ERBB3, the more sensitive it is to ATM kinase inhibitors. The mRNA expression was negatively correlated with afatinib sensitivity. By analyzing the relationship between these risk model genes and anticancer drug susceptibility, the results could provide a valuable reference for clinicians in clinical medication.

### Verification of Protein Expression of Model Genes Between KIRC Tissues and Normal Tissues

**FIGURE 7:**
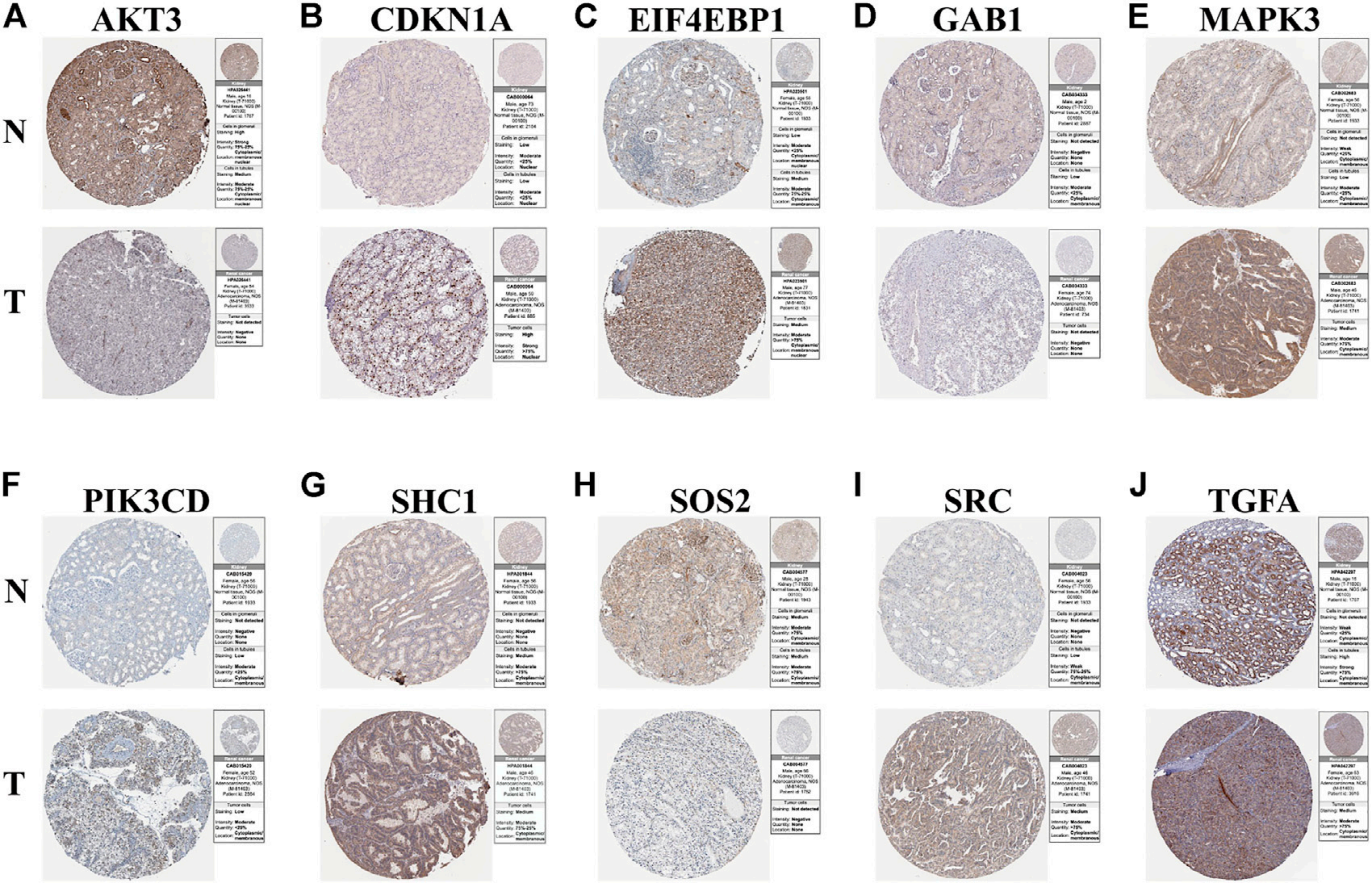
Results of immunohistochemistry. **(A–J)** Immunohistochemical images from the HPA database showing AKT3, CDKN1A, EIF4EBP1, GAB1, MAPK3, PIK3CD, SHC1, SOS2, SRC, and TGFA protein expressions in kidney tissues (N) and KIRC (T) tissues.

To study the protein expression levels of these risk model genes in KIRC, we explored the expression of AKT3, CDKN1A, EIF4EBP1, GAB1, MAPK3, PIK3CD, SHC1, SOS2, SRC, and TGFA in normal kidney and KIRC tissues from the HPA website (Figures 7A–J). The results show that the protein expression levels of CDKN1A, EIF4EBP1, MAPK3, PIK3CD, SHC1, SRC, and TGFA in KIRC tissues are higher than those in normal kidney tissues. Through further mechanistic research and clinical validation of these risk model genes in KIRC, they may be potential targets for the future treatment of KIRC. Afterward, to increase the credibility of our results, we validated the mRNA expression of these risk model genes using clinical specimens collected in the clinic (Supplementary Figures S1A–L). At last, to verify the reliability of the risk model in other databases, we used the ICGC database for validation. The validated results showed that the risk model also had a high predictive value in the ICGC database. The risk model could successfully classify patients in ICGC into high- and low-risk groups with significant prognostic differences (*p* = 0.00413). The 3-year AUC value of the ROC curve was 0.707, suggesting that the model has good predictive accuracy in ICGC (Supplementary Figures S2A–C).

## Discussion

In the past decades, studies on the relationship between the ERBB pathway and tumors have been ongoing; however, we lack direct evidence on the preciseness of the results. Most researchers believe that activating the ERBB pathway is essential for promoting tumorigenesis and development (Wang, 2017). The ERBB signaling pathway was found to be closely related to tumor immunity. Previous studies have found that immune cells and their extracellular factors play essential roles in anticancer immunity (Lai et al., 2021). Cancer-associated fibroblasts can limit the recruitment of immune effector cells, such as CD8^+^ T cells, to tumor tissues by secreting different chemokines (Mao et al., 2021). A recent study showed that programmed death-ligand 1 (PD-L1) expression and T-cell infiltration in patients with EGFR–mutant non-small cell lung cancer are related to immunotherapy (Chen et al., 2020). This suggests that the ERBB pathway affects cancer development through tumor immunity. In KIRC, EGFR overexpression is considered an extremely vital factor in the occurrence and development of RCC. The membranous expression of EGFR is related to high nuclear grade and poorly differentiated tumors (Ahel et al., 2015). In addition, the VHL-HIF-2α axis induces SET And MYND domain-containing 3 (SMYD3) upregulation, thereby activating EGFR to promote RCC progression (Liu et al., 2020). Furthermore, overexpression of EGFR and ERBB-2 is associated with the dedifferentiation and metastasis of RCC (Stumm et al., 1996). In the present study, we aimed to integrate the related genes in the ERBB pathway and establish a prognostic model for related KIRC patients.

AKT3 consists of two splice variants, Akt3 + s472 and Akt3 − s472, which mainly exist in the nerve cells and testes (Suyama et al., 2018). AKT/PKB plays a vital role in cell proliferation and apoptosis. After knocking out AKT3 in bladder cancer and lung cancer, the mitochondrial oxygen consumption in cancer cells rapidly decrease, indicating that AKT3 plays a vital role in the normal respiration of cancer cells (Kim et al., 2016). In addition, Akt3 plays an important role in brain development (Konishi et al., 1995). CDKN1A mainly regulates the cell cycle and DNA damage repair, affecting the occurrence and development of non-small cell lung cancer (Wu and Levine, 1997; Zamagni et al., 2020). Furthermore, CDKN1A participates in the occurrence and burgeoning of multiple myeloma along with p53 (Drozdkova et al., 2020). In previous studies, EIF4EBP1 was involved in tumor occurrence, invasion, and drug resistance (D’Abronzo and Ghosh, 2018). In KIRC, BRDT reduces the expression of c-MYC in RCC by regulating EIF4EBP1 and further enhances BRDT-targeted treatment RCC by regulating EIF4EBP1 or c-MYC (Wan et al., 2020). In addition, EIF4EBP1 is associated with the progression and poor prognosis of patients with liver tumors (Cha et al., 2015). ERBB3 encodes a transmembrane receptor tyrosine kinase composed of four domains (Kraus et al., 1989). The ERBB3 receptor has strong resistance to the pharmacological inhibition of EGFR and HER2 receptor tyrosine kinases in tumors (Eliseev et al., 2021). It was associated with GAB1 in the tumor proliferation and metastasis of head and neck squamous cell carcinoma and colorectal cancer (Seiden-Long et al., 2008; Hoeben et al., 2013). GAB1 promotes BRCA metastasis by interacting with the critical component PAR3 of the PAR complex and EMT of mammary gland tumors. In chronic liver injury, GAB1 plays an essential role in inhibiting apoptosis and reducing liver injury, fibrosis, and tumorigenesis (Mizutani et al., 2021).

Mitogen-activated protein kinase was considered a serine/threonine-protein kinase, which mainly exists in mammals. It is associated with cell proliferation, differentiation, inflammation, apoptosis, and various physiological and pathological processes (Yu et al., 2020). MicroRNA-143 regulates the proliferation and bone metastasis of human BRCA cells by targeting MAPK3 (Du et al., 2020). MAPK1/MAPK3 kinase can also reduce mitochondrial autophagy through ULK1 degradation and promote bone metastasis in BRCA (Deng et al., 2021). PIK3CD encodes the phosphatidylinositol 3-kinase (PI3K) catalytic subunit P110 δ related to cancer, and PIK3C deactivates the AKT/GSK-3 β/β-catenin signaling pathway, promoting the occurrence and development of colorectal cancer (Chen et al., 2019). The long-chain noncoding RNA PIK3CD-AS2 promotes the occurrence and evolution of lung adenocarcinoma by inhibiting the YBX1–mediated p53 pathway, suggesting that PIK3CD-AS2 is a crucial regulator of LUAD (Zheng et al., 2020). SHC1 is an essential molecule that DEPDC1B regulates the evolution of bladder cancer progression. SHC1 knockout can reduce the effect of DEPDC1B on bladder cancer induction (Lai et al., 2020). Furthermore, SHC1 can regulate PTRF expression through certain pathways associated with the occurrence and evolution of KIRC (Zhao et al., 2020). SRC regulates tumorigenesis and angiogenesis through related signal transduction pathways. The activation of c-SRC (SRC) induces EMT, leading to the development of pancreatic cancer (Nagathihalli and Merchant, 2012). SRC mediates the activation of receptor tyrosine kinases and constitutes another bypass mechanism of transactivation after drug inhibition. This bypass mechanism is essential for colorectal cancer cells to develop resistance to EGFR-targeted therapy (Gargalionis et al., 2014). MALAT1 can increase the expression of TGFA and promote the proliferation and metastasis of osteosarcoma by inhibiting mir376a (Luo et al., 2016). MiR-137 controls the occurrence and development of non-small cell lung cancer by regulating TGFA (Liu et al., 2017).

RCC is a highly complicated process that is controlled by multiple target genes. With the increase in research, there are many related prognostic models in KIRC patients (Xu et al., 2020b; Wu et al., 2021). This study mainly used the LASSO regression analysis to establish a risk model associated with the ERBB signaling pathway for KIRC patients. LASSO regression is a compressed estimation. It obtains a more refined model by constructing a penalty function, compressing some coefficients, and setting some coefficients to zero. It can realize the selection of variables simultaneously as parameter estimation, to better solve the multicollinearity problem in the regression analysis, and explain the results well. However, the disadvantages are also obvious. Compressing some coefficients will cause underfitting of the model, and it is not easy to calibrate. The current study successfully used SHC1, GAB1, SOS2, SRC, AKT3, EREG, EIF4EBP1, ERBB3, MAPK3, TGFA, CDKN1A, and PIK3CD in the ERBB pathway to establish a risk model for predicting the prognosis of KIRC patients. We drew 5-, 7- and 10-year ROC curves based on the risk model. In general, the AUC value was greater than 0.7, indicating that the risk model has high prediction accuracy. In general, our prognostic model has higher prediction accuracy than other prognostic models and is also a supplement to other prognostic models. However, LASSO regression has limitations in gene-selection research. The problem with model interpretation ability is that many variables in a multiple linear regression model may be independent of the response variables. Multicollinearity can be produced when there is an apparent correlation between multiple prediction variables. These situations increase the complexity of the model and weaken its interpretative abilities. At present, variable selection is required; however, our prognostic model may provide a more comprehensive and personalized treatment for KIRC patients.

## Conclusion

Our study identified that the 12 genes used to build prognostic risk models were studied to varying degrees in a variety of tumors. However, some risk model genes have not been intensively studied in KIRC. Therefore, they must be closely monitored in future studies.We believe that our established prognostic risk model related to the prognosis of KIRC patients can provide individualized treatment options for future diagnosis and treatment.

## Data Availability

The raw data supporting the conclusion of this article will be made available by the authors, without undue reservation.

## Abbreviations

ACC, Adrenocortical carcinoma; AKT3, AKT serine/threonine kinase 3; BRCA, Breast invasive carcinoma; BLCA, Bladder urothelial carcinoma; ccRCC, Clear cell renal cell carcinoma; CDKN1A, Cyclin-dependent kinase inhibitor 1A; CNV, Copy number variation; ERBB3, Erb-B2 receptor tyrosine kinase 3; EIF4EBP1, Eukaryotic translation initiation factor 4E binding protein 1; EREG, Epiregulin; GAB1, GRB2-associated binding protein 1; GSEA, Gene set enrichment analysis; HPA, Human Protein Atlas; HR, hazard ratio; KIRC, Kidney renal clear cell carcinoma; LASSO, Least absolute shrinkage and selection operator; MAPK3, Mitogen-activated protein kinase 3; OS, Overall survival; PIK3CD, Phosphatidylinositol-4,5-bisphosphate 3-kinase catalytic subunit delta; PPI, Protein–protein interaction; ROC, Receiver operating characteristic; SHC1, SHC adaptor protein 1; SNV, Single-nucleotide variation; SOS2, SOS Ras/Rho guanine nucleotide exchange factor 2; SRC, SRC proto-oncogene, non-receptor tyrosine kinase; TCGA, The Cancer Genome Atlas; TGFA, Transforming growth factor-alpha.
