# Supplementary figures and images for: The Role of ERBB Signaling Pathway-Related Genes in Kidney Renal Clear Cell Carcinoma and Establishing a Prognostic Risk Assessment Model for Patients
Source: Front Genet. 2022 Jul 12;13:862210. doi: 10.3389/fgene.2022.862210 (PMC9314565; doi:10.3389/fgene.2022.862210)

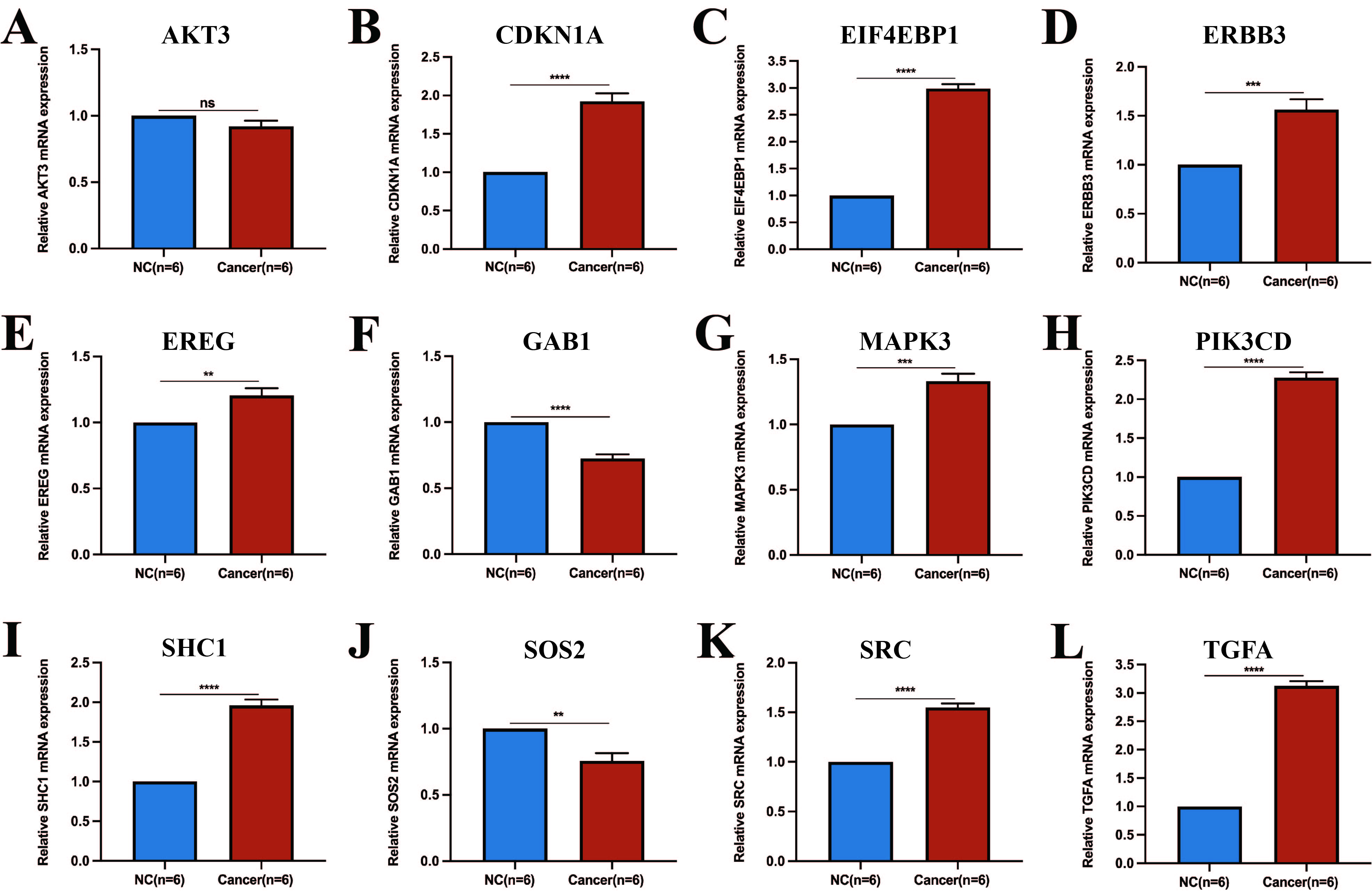

Supplement: Supplementary file 1 [file Image1.JPEG]

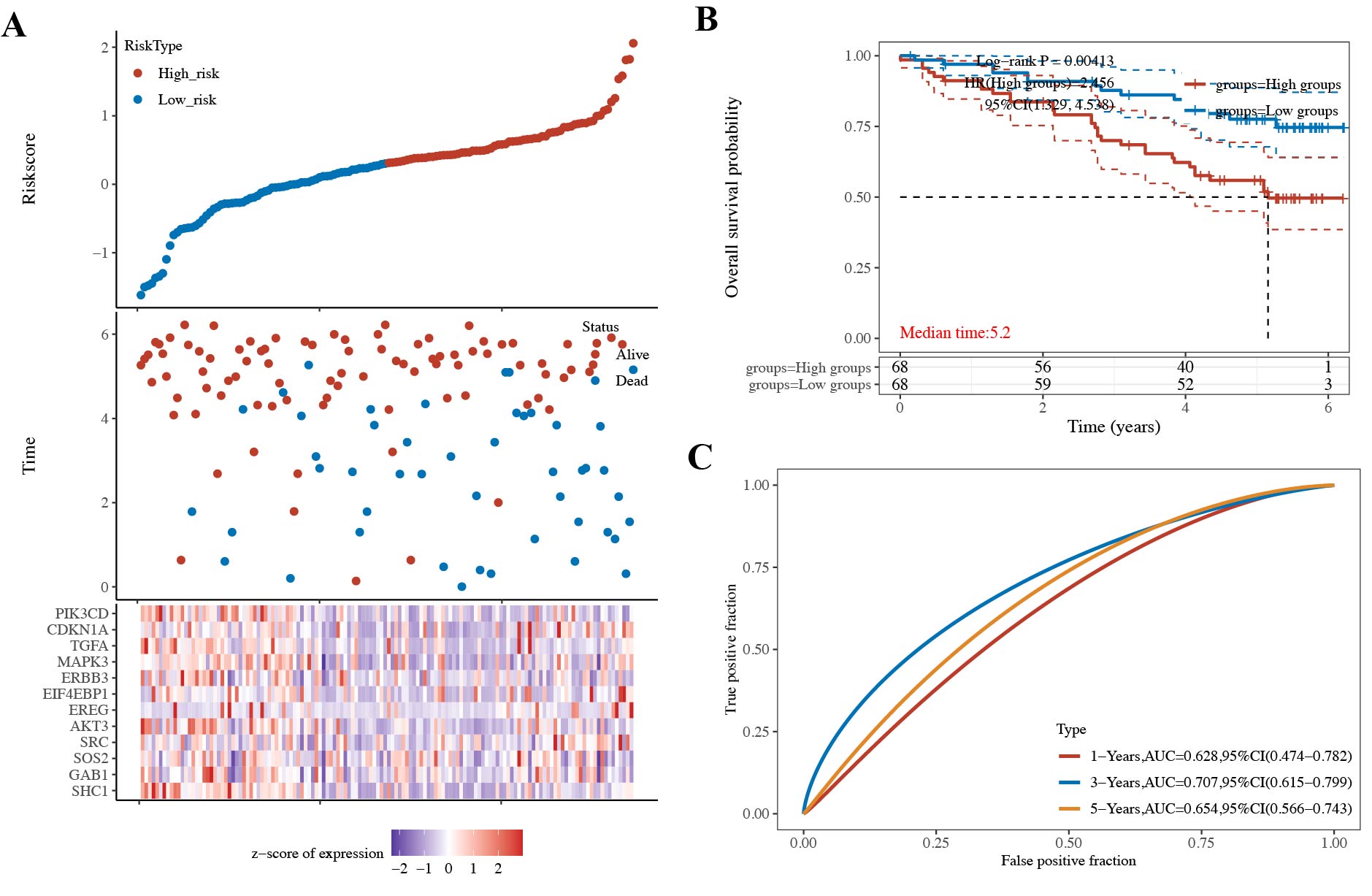

Supplement: Supplementary file 2 [file Image2.JPEG]
