# Supplementary material for: The Role of ERBB Signaling Pathway-Related Genes in Kidney Renal Clear Cell Carcinoma and Establishing a Prognostic Risk Assessment Model for Patients
Source: Front Genet. 2022 Jul 12;13:862210. doi: 10.3389/fgene.2022.862210 (PMC9314565; doi:10.3389/fgene.2022.862210)
Supplement: Supplementary file 3 [file DataSheet1.PDF]

Supplementary Materials

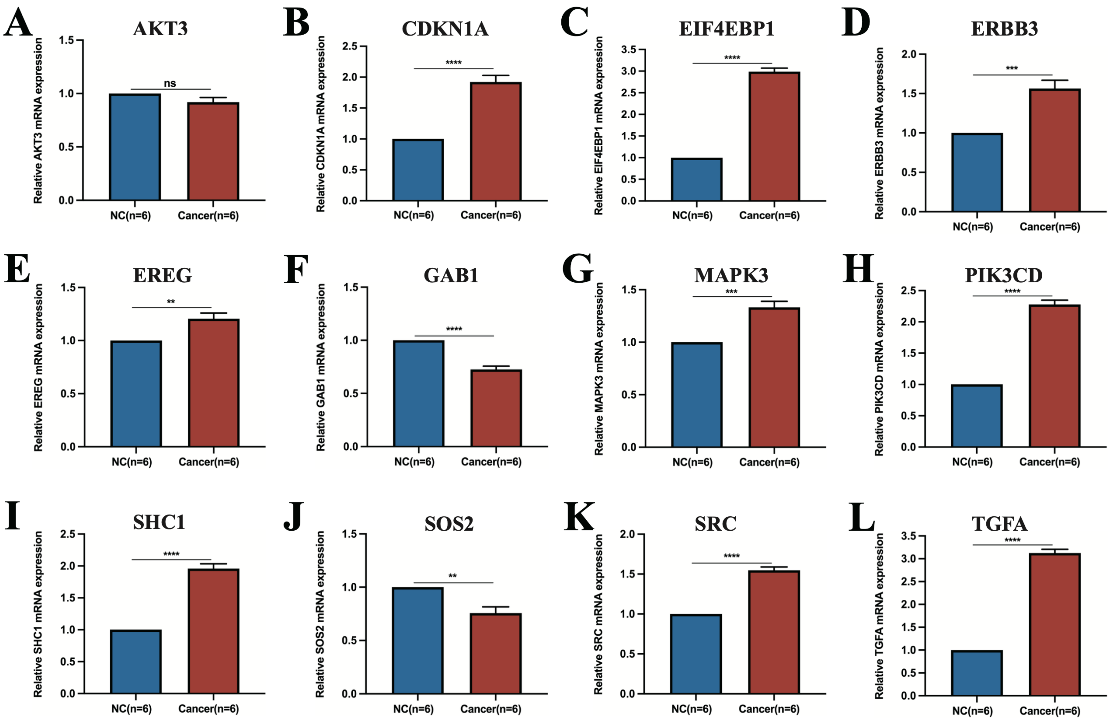

Figure S1. The mRNA expression results of risk model genes were detected in KIRC pathological tissues and normal control tissues. (A) AKT3; (B) CDKN1A; (C) EIF4EBP1; (D) ERBB3; (E) EREG; (F) GAB1; (G) MAPK3; (H) PIK3CD; (I) SHC1; (J) SOS2; (K) SRC; and (L) TGFA. $**p < 0.01$, $***p < 0.001$, $****p < 0.0001$, ns means no significance.

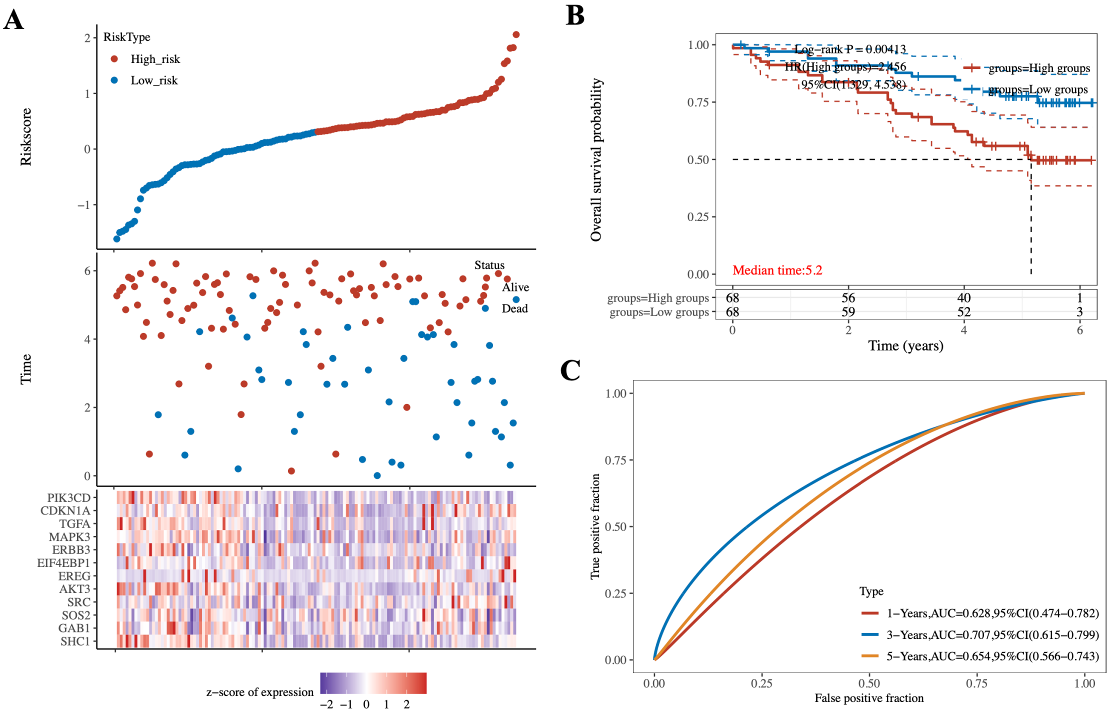

Figure S2. Validation of the risk model was performed using the ICGC database. (A) Multivariate Cox regression analysis was used to construct a predictive model. (B) Kaplan-Meier curves show the overall survival of patients in the high and low-risk groups in the ICGC database (p = 0.00413). (C) The timeROC analysis discriminated against the accuracy of the predictive model.
